# The relationship between urinary albumin-to-creatinine ratio and bacterial profiles/inflammatory markers in diabetic foot infections: a retrospective cross-sectional observational study

**DOI:** 10.3389/fendo.2026.1724201

**Published:** 2026-04-10

**Authors:** Hao Wang, Fan Zhang, Gui-Ling Sun, Hui-Min Xu, Shao-Na Niu, Yan Peng

**Affiliations:** 1 School of Clinical Medicine, Shandong Second Medical University, Weifang, Shandong, China; 2 Department of Endocrinology and metabolism, Linyi People’s Hospital, Shandong Second Medical University, Linyi, Shandong, China

**Keywords:** bacterial profiles, diabetic foot infection, diabetic nephropathy, inflammation, Wagner classification

## Abstract

**Background:**

Diabetic foot disease (including diabetic foot infection, DFI) and diabetic nephropathy (DN) are common diabetic complications. Patients with proteinuric DN are more likely to develop DFI, but data linking proteinuria and DFI are limited. We reviewed Urinary Albumin-to-Creatinine Ratio (UACR), pathogenic bacteria and inflammatory indicators of DFI patients from a northern Chinese tertiary hospital (2020–2023).

**Methods:**

We analyzed clinical data from 325 DFI patients, grouping them by UACR: normoalbuminuria (UACR < 30 mg/g), microalbuminuria (30 ≤ UACR < 300 mg/g), and macroalbuminuria (UACR ≥ 300 mg/g).

**Results:**

This is a single-center, retrospective cross-sectional observational study conducted at a tertiary hospital in northern China. We analyzed the association between UACR and the characteristics of DFI, and adjusted for potential confounding variables in the regression analysis, including glycemic control status, Wagner classification and peripheral vascular disease. This study included 325 DFI patients (66.8% male; 33.2% female), with average diabetes duration 11.9 ± 7.62 years and DFI duration 2.29 ± 0.35 months. We identified 447 bacterial isolates from secretions (193 Gram-positive, 241 Gram-negative, 13 fungi); 67.69% had single-bacterial infections, 32.31% polymicrobial. Bacteria types differed by UACR: normoalbuminuria group had mostly Gram-positive (55.04%, 50.26% Staphylococcus aureus); microalbuminuria group had more polymicrobial infections (40.71%) and dominant Gram-negative (61.21%); macroalbuminuria group also had more Gram-negative (58.57%). Higher UACR correlated with worse inflammation and metabolism.

**Conclusion:**

DFI patients with different UACR levels have distinct pathogenic bacteria. Higher UACR relates to worse inflammation and metabolic issues, suggesting a link between DN and DFI. Stratifying by UACR shows local DFI pathogen distribution, guiding clinicians’ initial antibiotic use.

## 1 Introduction

The prevalence of diabetes mellitus is growing in China and worldwide. According to statistics, the age-standardized diabetes prevalence in 2022 was 14.1%, with approximately 828 million adults having diabetes—representing a significant increase from 630 million in 1990. Among these cases, India (212 million) and China (148 million) accounted for the largest proportions (1).Diabetic foot ulcer(DFU) is a serious complication for diabetes, usually caused by neuropathy and/or peripheral artery disease (2). The prevalence of global DFU is about 6.3% (5.4-7.3%) in a meta-analysis published in 2017 (3). The prevalence rate of DFU is about 8.1%,in China (4). It is predicted that nearly half of diabetic patients with DFU will develop DFI (5). DFI remains the most frequent complication requiring hospitalization and the most common reason leading to lower extremity amputation (6). DN and DFI are common complications of diabetes. The prevalence of DN is about 21.8% of patients with type 2 diabetes according to a meta-analysis (7).

UACR ≥ 30 mg/g is the core marker of early kidney injury. It corrects for fluctuations in urine volume via urinary creatinine, allowing a single random urine test (such as early-morning midstream urine) to suffice (8). It is not affected by muscle mass or diet and can indicate changes in kidney function even when eGFR is normal. UACR reflects damage to the glomerular filtration membrane. It shares pathological mechanisms (such as oxidative stress and inflammation) with systemic microvascular endothelial dysfunction and, at the same time, causes increased arterial wall stiffness, which indirectly supports the predictive value of UACR for DFI (9). On the other hand, in a high-glucose environment, neutrophils become abnormally activated. Neutrophils with impaired function release excessive amounts of Neutrophil extracellular traps (NETs), which in turn exacerbates the progression of inflammation and damage to kidney function (10). Gupta et al. have shown that in patients with CKD, UACR levels are significantly associated with inflammatory status, and this association is independent of kidney function (eGFR): the higher the UACR, the higher the levels of inflammatory markers (11).

However, the direct association between the proteinuria status represented by UACR and DFI has not been clearly established, and there is also a lack of systematic research on their mutual influence in terms of pathogen distribution and inflammatory severity. Based on the aforementioned research status, our study aims to clarify the distribution characteristics of pathogens and the variation patterns of inflammatory markers in patients with DFI across different UACR levels. This will be achieved by conducting a retrospective analysis of clinical data from DFI patients treated in a tertiary hospital in northern China during 2020–2023, with UACR used as the stratification basis. The study thereby intends to provide evidence-based support for the precision diagnosis and treatment of DFI (such as empirical antibiotic selection and inflammatory monitoring) and address the gap in current research on the association between proteinuria and DFI.

## 2 Subjects and methods

### 2.1 Subjects

This was a retrospective cross-sectional observational study of subjects with DFI who were treated in the tertiary hospital in northern China between January 2020 and December 2023. They all understood the purpose of the study, signed the informed consent form, and received approval from the local ethics committee. We identified pathogenic microorganisms through microbial culture and sample analysis. All pus swabs were collected after patient admission and prior to the initiation of antibiotic therapy. Professionally trained nurses cleaned the wound surface and its surrounding skin with sterile normal saline, then collected secretions from the wound base using sterile cotton swabs. Collected specimens were placed in sterile test tubes, immediately transported to the microbiology laboratory of our hospital, and the laboratory performed strain culture, isolation, and species identification on the specimens using standard microbiological methods.

The Wagner wound classification system was used to evaluate each patient’s ulcer. Grade 0 (Pre-ulcerative/high-risk foot); Grade 1 (Superficial ulcer involving epidermis, dermis, or subcutaneous tissue); Grade 2 (ulcer deep to tendon, bone, or joint); Grade 3 (ulcer with abscess or osteomyelitis or osteomyelitis with infection); Grade 4 (Forefoot gangrene); Grade 5 (Whole foot gangrene). To control the impact of wound severity on pathogen distribution and inflammatory levels, we conducted a subgroup analysis of the Wagner classification, which was categorized into mild-to-moderate, severe, extremely severe. Stratify patients by UACR levels (UACR <30 mg/g, 30≤UACR<300 mg/g, UACR ≥300 mg/g). Use intra-group comparisons to reduce confounding from differences in kidney function. The presence of age, gender, duration of the disease, smoking history, alcohol consumption history, related complications, and laboratory indicators: C-peptide (C-P), glycated hemoglobin (HbA1c), white blood cell (WBC), neutrophils (NEUT), procalcitonin (PCT), erythrocyte sedimentation rate (ESR), c-reactive protein(CRP), neutrophils ratio (NEUT), platelet (PLT), lymphocyte (LYM), serum albumin (ALB), triglyceride (TG), total cholesterol (TC), low-density lipoprotein cholesterol (LDL-C), and hemoglobin (Hb), history of foot ulceration, foot ulcer localization, Laboratory examination and radiologic evaluations were collected from the Hospital Information System. Excluded patients who have used antibiotics or immunosuppressants in the past two weeks, those with missing key data, and Wagner grade 0 patients (since high-risk feet lack clear infection assessment criteria), to ensure all included patients are confirmed DFI cases. Examinations of patients’ pain sensation, vibration sensation, tactile sensation, and other sensory functions were performed using disposable syringe needles, tuning forks, nylon monofilaments, and other tools to determine the presence or absence of peripheral neuropathy; peripheral vascular disease was diagnosed based on color Doppler ultrasound of blood vessels and changes in dorsal pedal artery pulsation.

### 2.2 Statistical analysis

Normality test is performed via the Kolmogorov-Smirnov test (K-S test).Normally distributed variables are expressed as mean ± standard deviation, and one-way analysis of variance (ANOVA) is used for comparisons between multiple groups, followed by the Student-Newman-Keuls (SNK) method for *post-hoc* tests. Non-normally distributed variables are expressed as median (interquartile range), and the Kruskal-Wallis test is used for comparisons between multiple groups, with subsequent adjustment of the significance level using the non-parametric *post-hoc* Bonferroni method. For categorical variables, when the chi-square test is used for multiple group comparisons, adjacent categories (e.g., Wagner grades) are merged to reduce the number of comparisons, which indirectly lowers the risk of multiple comparisons. Additionally, multivariate analysis of polymicrobial infections and inflammatory markers was performed using multivariate logistic regression. P-values less than 0.05 are considered statistically significant.

## 3 Result

### 3.1 Baseline data of diabetic foot patients

This retrospective study analyzed 325 patients with culture-positive DFI treated at a tertiary hospital in northern China between 2020 and 2023. The cohort comprised 217 males (66.8%) and 108 females (33.2%), with a mean diabetes duration of 11.9 ± 7.62 years and a mean DFI duration of 2.29 ± 0.35 months. Patients were stratified into three groups based on urinary UACR thresholds for DN: UACR < 30 mg/g, 30 ≤ UACR <300 mg/g, and UACR ≥ 300 mg/g. Statistical analysis revealed no significant associations between UACR categories and demographic or clinical factors, including age, sex, hypertension, peripheral neuropathy, peripheral vascular disease, smoking, or alcohol use (p> 0.05).

Wagner grades were categorized into three severity tiers: mild-to-moderate (grades 1-2) as group 1, severe (grade 3)as group 2, and critical (grades 4-5)as group 3. Comparative analysis showed that the proportion of patients with a Urinary Albumin-to-Creatinine Ratio (UACR) ≥ 30 mg/g in the severe Diabetic Foot Infection (DFI) group was significantly higher than that in the mild DFI group (**Table 1**).

**Table 1:** Initial characteristics of the whole population, comparison between the Normoalbuminuria group (UACR <30 mg/g), Microalbuminuria group (30 ≤ UACR <300 mg/g) and Macroalbuminuria group (UACR ≥300 mg/g).

Parameters		UACR<30	30≤UACR<300	UACR≥300	F/Χ^2^	P
Sex	Male	70(66.7%)	76(67.3%)	71(66.4%)	0.021	0.990
	Female	35(33.3%)	37(32.7%)	36(33.6%)		
Age(years)		64.72±11.06	64.93±11.11	61.90±12.45	2.322	0.100
Wagner group	Group 1	69(65.7%)	67(59.3%)	61(57.0%)	8.731	0.068
	Group 2	27(25.7%)	25(22.1%)	21(19.6%)		
	Group 3	9(8.6%)	21(18.6%)	25(23.4%)		
HbA1c (%)	>7	87(82.9%)	104(92.0%)	99(92.5%)	6.571	0.037
	≤7	18(17.1%)	9(8.0%)	8(7.5%)		
Hypertension	No	66(62.9%)	62(54.9%)	61(57.0%)	1.514	0.469
	Yes	39(37.1%)	51(45.1%)	46(43.0%)		
Peripheral neuropathy	No	43(41.0%)	32(28.3%)	40(37.4%)	4.012	0.134
	Yes	62(59.0%)	81(71.7%)	67(62.6%)		
Peripheral vascular disease	No	25(23.8%)	22(19.5%)	28(26.2%)	1.436	0.488
	Yes	80(76.2%)	91(80.5%)	79(73.8%)		
Smoking	No	81(77.1%)	83(73.5%)	70(65.4%)	3.793	0.150
	Yes	24(22.9%)	30(26.5%)	37(34.6%)		
Drinking	No	78(74.3%)	87(77.0%)	82(76.6%)	0.254	0.881
	Yes	27(25.7%)	26(23.0%)	25(23.4%)		
total		105	113	107		

Age was analyzed using ANOVA for comparisons between multiple groups, while other Parameters were analyzed using the chi-square test.

Significant p-values are indicated by underscores.

F, ANOVA; Χ^2^, chi-square test.

### 3.2 Characteristics of bacterial infections in patients with DFI with different UACR grades

**Figure 1:**
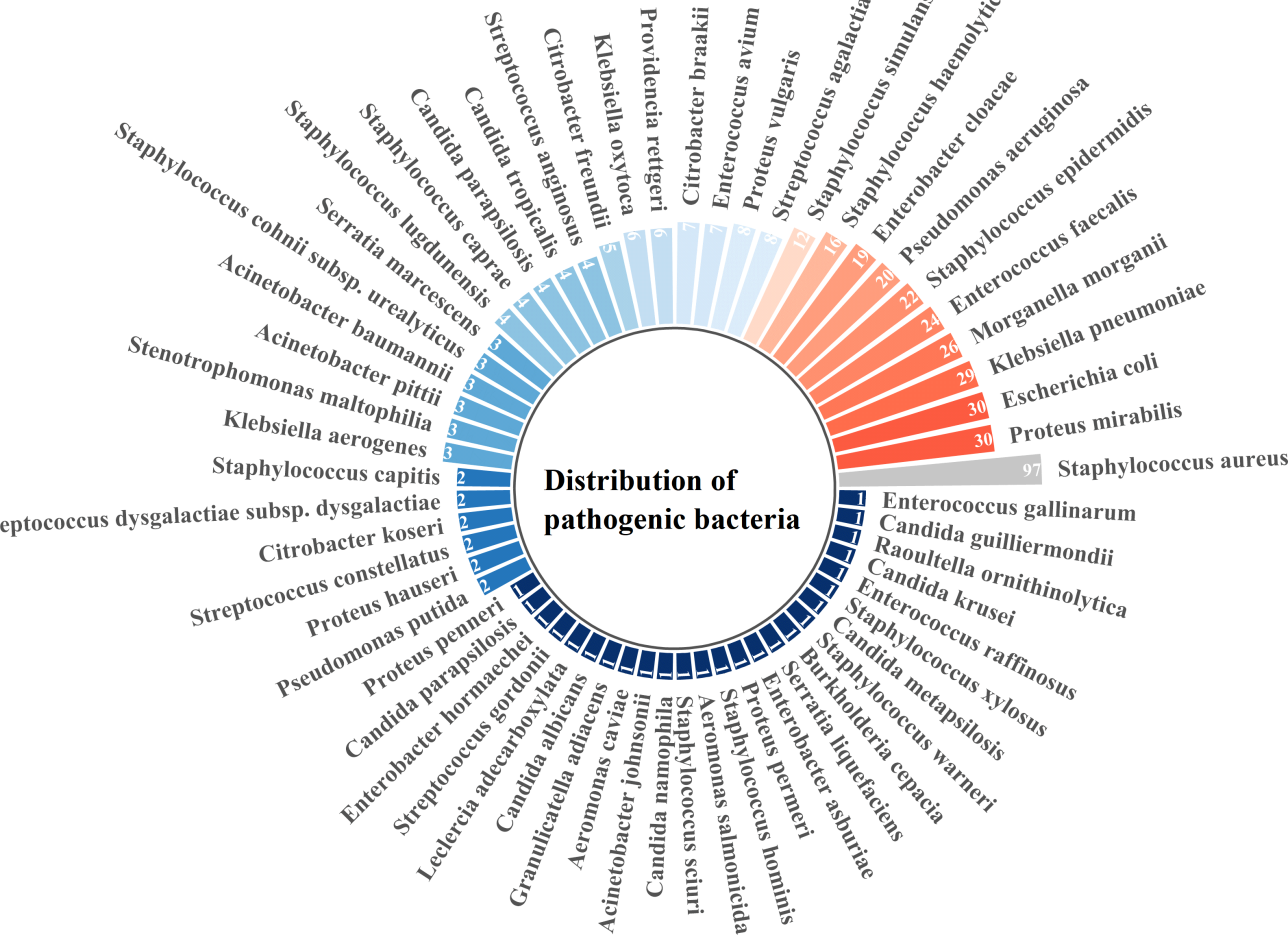
Distribution of pathogenic bacteria isolated from ulcer secretions in 325 patients with DFI. A total of 447 pathogenic strains were detected, including 193 Gram-positive bacteria, 241 Gram-negative bacteria and 13 fungi. The number and percentage of each type of pathogen are labeled on the chart. DFI, diabetic foot infection.

**Figure 2:**
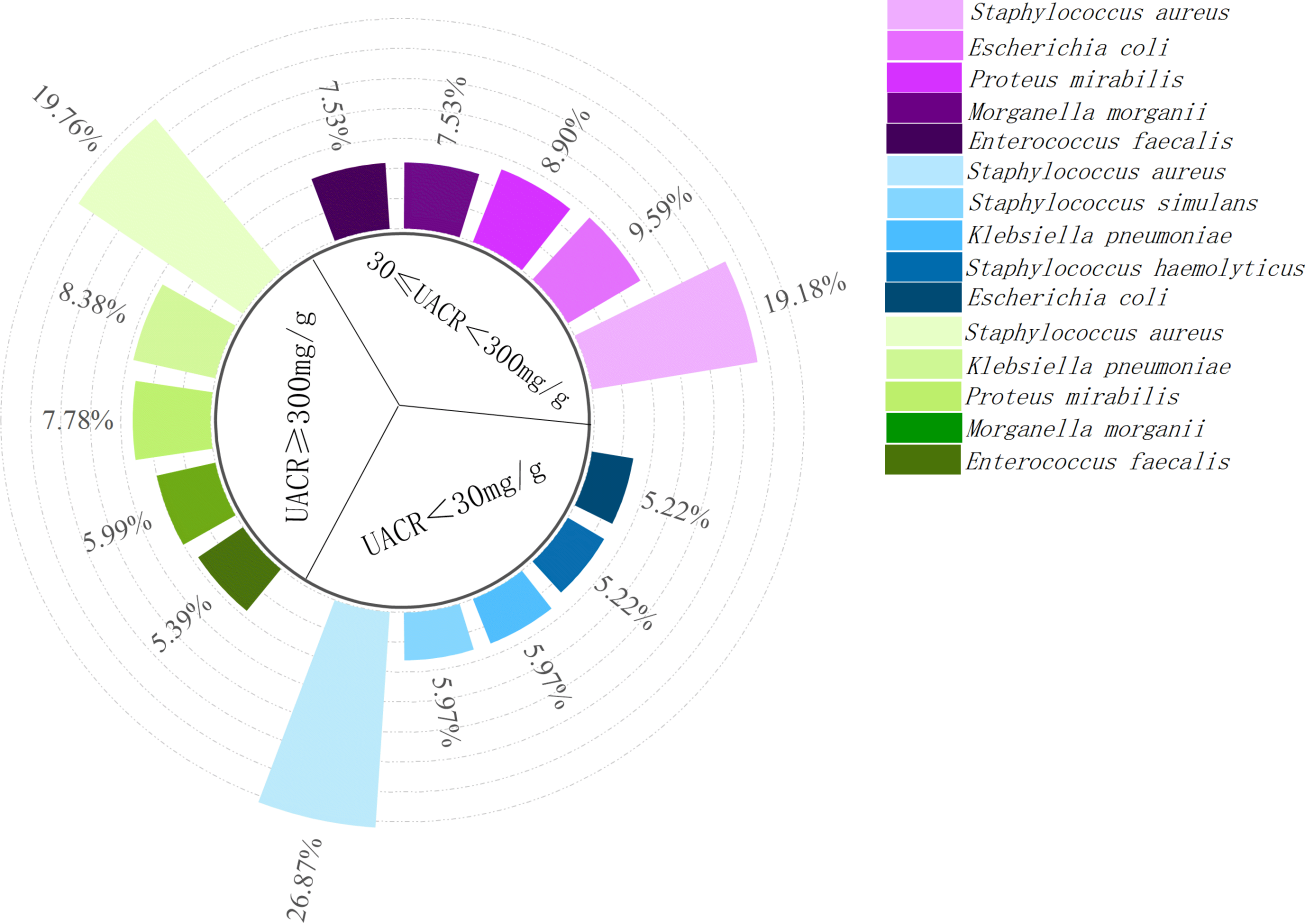
Distribution of the top 5 pathogenic bacteria in DFI patients stratified by UACR. Staphylococcus aureus was the most prevalent pathogen in all three UACR groups, with the detection rates of 26.87%, 19.18% and 19.76% in the UACR <30mg/g,30≤UACR<300mg/g and UACR≥300mg/g groups,respectively.UACR <30mg/g:normoalbuminuria; 30≤UACR<300mg/g:microalbuminuria; UACR≥300mg/g: macroalbuminuria. DFI, diabetic foot infection; UACR, urinary albumin-to-creatinine ratio.

**Figure 3:**
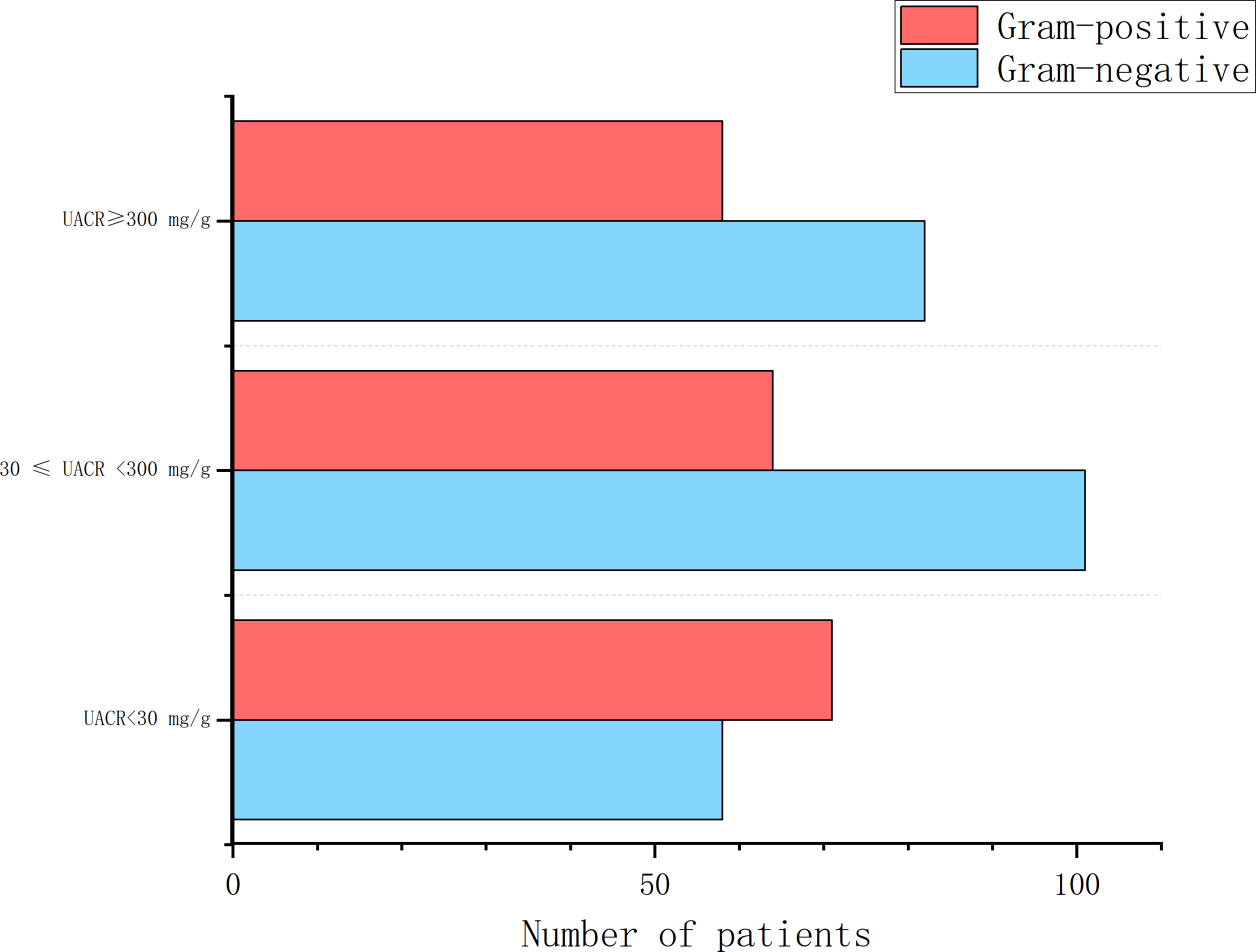
Gram-stain profiles of bacterial pathogens in DFI patients stratified by UACR levels. There was a statistically significant difference in the distribution of Gram-stain types among the three groups (p=0.014). The normoalbuminuria group was predominantly infected with Gram-positive bacteria (55.04%), while the microalbuminuria and macroalbuminuria groups were dominated by Gram-negative bacteria (61.21% and 58.57%,respectively). DFI, diabetic foot infection; UACR, urinary albumin-to-creatinine ratio.

**Figure 4:**
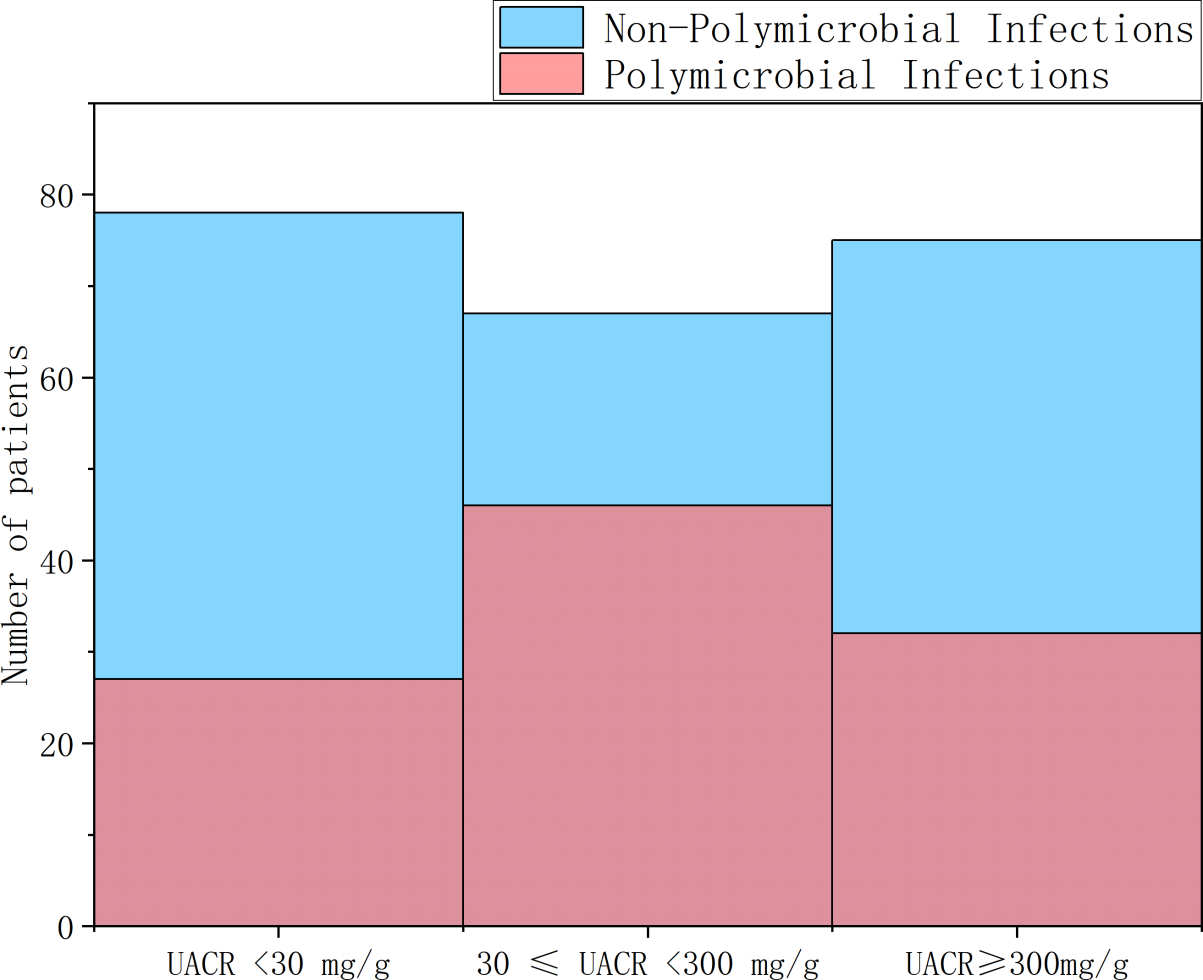
The rate of polymicrobial infections in DFI patients with different UACR levels. The probability of polymicrobial infections increased with the elevation of UACR (p=0.049), and the microalbuminuria group had the highest polymicrobial infection rate (43.81%, 46/105). DFI, diabetic foot infection; UACR, urinary albumin-to-creatinine ratio.

A total of 447 strains of pathogenic bacteria were detected in secretions culture from 325 patients, including 193 gram-positive bacteria, 241 gram-negative bacteria, and 13 fungi (**Figure 1**). There were 18 multi-drug resistant strains of Gram-positive bacteria, with a multi-drug resistance rate of 9.32%, and 28 multi-drug resistant strains of Gram-negative bacteria, with a multi-drug resistance rate of 11.62%.Among the 447 pathogens detected, the top 5 pathogens were Staphylococcus aureus (97, 21.80%), Proteus mirabilis (30, 6.74%), Escherichia coli (30, 6.74%), Klebsiella pneumoniae (30, 6.52%), and Morganella morgani (26, 5.84%). Among them, Staphylococcus aureus ranked first in all three groups of UACR classification (**Figure 2**). Based on the UACR grading, from low to high, Staphylococcus aureus accounted for 19.18% (28/146), 19.76% (33/167), and 26.87% (36/134) of the detected pathogens, respectively. The results indicated that there was a statistically significant difference in bacterial staining among the three UACR grading groups (p=0.014)(**Figure 3**). When UACR was less than 30mg/g, the majority of the infecting bacteria were Gram-positive (55.04%, 71/129). Once UACR reached or exceeded 30mg/g, the number of Gram-negative bacteria significantly surpassed that of Gram-positive bacteria. Specifically, in the 30≤UACR<300mg/g and UACR≥300mg/g groups, the proportions were 61.21% (101/165) and 58.57% (82/140).As UACR increases, the probability of polymicrobial infection also rises (p=0.049), particularly in the group with 30≤UACR<300mg/g (43.81%, 46/105). Compared to the other two groups, the proportion of patients with polymicrobial infection is higher (**Figure 4**).

We performed a logistic regression analysis with polymicrobial infection as the dependent variable, adjusting for such risk factors as Wagner classification, UACR classification, HbA1c achievement status, smoking history, alcohol consumption history, peripheral vascular disease, and peripheral neuropathy. The results showed that Wagner classification and UACR classification were independently associated with polymicrobial infection(**Table 2**).

**Table 2:** The independent variables for polymicrobial infection was assessed by logistic regression analysis.

Variables	B	SE	OR	P	95%CI
UACR <30 mg/g	-	-	1.00	-	
30≤UACR<300mg/g	0.664	0.314	2.029	0.034	1.050 ~ 3.593
UACR≥300mg/g	0.272	0.333	1.320	0.414	0.684 ~ 2.519
Wagner group 1	-	-	1.00	-	
Wagner group 2	1.071	0.305	2.918	0.001	1.606 ~ 5.301
Wagner group 3	1.029	0.328	2.797	0.002	1.472 ~ 5.315

Significant p-values are indicated by underscores.

B,regression coefficient; SE, standard error; OR, odd ratio.

### 3.3 Factors associated with patients with different UACR classification

Comparative analysis of clinical parameters across UACR groups revealed statistically significant differences (p < 0.05) in HbA1c, CRP, WBC, NEUT, Systemic Immune-Inflammation Index(SII), PCT, and ESR between the UACR <30 mg/g group and the other two groups, with these markers exhibiting an increasing trend as UACR levels rose. In the UACR ≥300 mg/g group, TG, LDL-C, TC, LYM, Hb differed significantly from the other groups. Specifically, TG, LDL-C, and TC were markedly elevated, whereas lymphocytes and Hb were reduced. Serum albumin levels also differed significantly across all three groups, demonstrating an inverse correlation with UACR progression (**Table 3**).

**Table 3:** Clinical and biochemical characteristics of DFI patients with different UACR group.

Factors	UACR<30(1)	30≤UACR<300(2)	UACR≥300(3)	F/H	P	P(1)(2)	P(1)(3)	P(2)(3)
HbA1c(%)	8.71±2.02	9.65±2.14	9.48±2.60	8.954	0.001	0.001	0.001	0.451
C-P(ug/L)	1.08(0.55~1.95)	0.94(0.46~1.91)	0.91(0.51~1.65)	1.728	0.422	1.000	0.144	0.043
TG (mmol/L)	1.16(0.92~1.57)	1.18(0.84~1.62)	1.31(1.03~1.87)	6.730	0.035	0.601	0.041	0.017
LDL-C (mmol/L)	2.51±0.87	2.43±1.10	2.96±1.18	7.555	0.003	0.436	0.008	0.002
TC (mmol/L)	4.02±1.12	3.89±1.40	4.63±1.46	9.223	0.001	0.370	0.004	0.001
ALB (g/L)	38.31±5.74	36.75±5.49	33.42±5.92	20.336	0.001	0.015	0.001	0.001
CRP (mg/L)	10.65(3.10~47.15)	26.2(5.95~91.00)	33.68(8.26~104.15)	12.074	0.002	0.010	0.005	1.000
ESR(mm/60min)	39.05±29.89	56.98±30.78	70.13±32.23	22.507	0.001	0.001	0.001	0.005
WBC(10^9/L)	7.53(5.86~9.90)	8.85(6.63~12.90)	8.43(6.49~15.98)	9.017	0.011	0.011	0.091	1.000
NEUT(10^9/L)	5.16(3.61~7.39)	6.41(4.48~10.91)	6.17(4.51~9.30)	10.090	0.006	1.000	0.014	0.022
PLT(10^9/L)	250.33±84.75	279.67±173.86	282.84±107.22	2.047	0.093	0.247	0.031	0.277
LYM(10^9/L)	1.69±0.76	1.64±0.67	1.45±0.65	3.479	0.006	0.837	0.005	0.007
SII	684.14	1046.90	1280.04	18.443	0.001	0.005	<0.001	0.891
		
	(403.87~1455.99)	(546.79~2363.67)	(679.74~2430.02)					
PCT(ng/ml)	0.05(0.03~0.09)	0.06(0.04~0.15)	0.07(0.04~0.24)	11.890	0.003	0.035	0.003	1.000
Hb(g/L)	124.82±19.22	121.65±19.30	110.57±20.48	15.125	0.001	0.239	0.001	0.001

Normally distributed variables were presented as mean ± standard, and comparisons between the two groups were conducted using an independent samples t-test. Abnormally distributed variables were presented as median (25th percentile~75th percentile), and comparisons between the two groups were performed using the Mann–Whitney U-test. Statistical differences were defined by P (twotailed) less than 0.05.

Significant p-values are indicated by underscores.

F, ANOVA; H, Kruskal-Wallis tests; C-P, C-peptide; HbA1c,glycated hemoglobin; WBC,white blood cell; NEUT, neutrophils; PCT, procalcitonin; ESR, erythrocyte sedimentation rate; CRP, c-reactive protein; NEUT, neutrophils ratio; PLT, platelet; LYM, lymphocyte; ALB, albumin; TG, triglyceride; TC, total cholesterol; LDL-C, low-density lipoprotein cholesterol; Hb, hemoglobin; SII, systemic Immune-Inflammation Index.

We further refined the logistic regression with UACR classification as the dependent variable and adjusted it according to factors related to DFI, including Wagner classification, HbA1c achievement status, smoking history, alcohol consumption history, peripheral vascular disease, and vascular disease. The results showed that ESR has more significant diagnostic value than other inflammatory markers in evaluating DFI combined with DN (**Table 4**).

**Table 4:** Characteristics of ESR was assessed by logistic regression analysis.

Variable	B	SE	OR	P	95%CI
ESR	0.028	0.005	1.028	0.001	0.018 ~ 0.039

B, regression coefficient; SE, standard error; OR, odd ratio.

### 3.4 Bacterial distribution characteristics in patients with different Wagner grades

**Figure 5:**
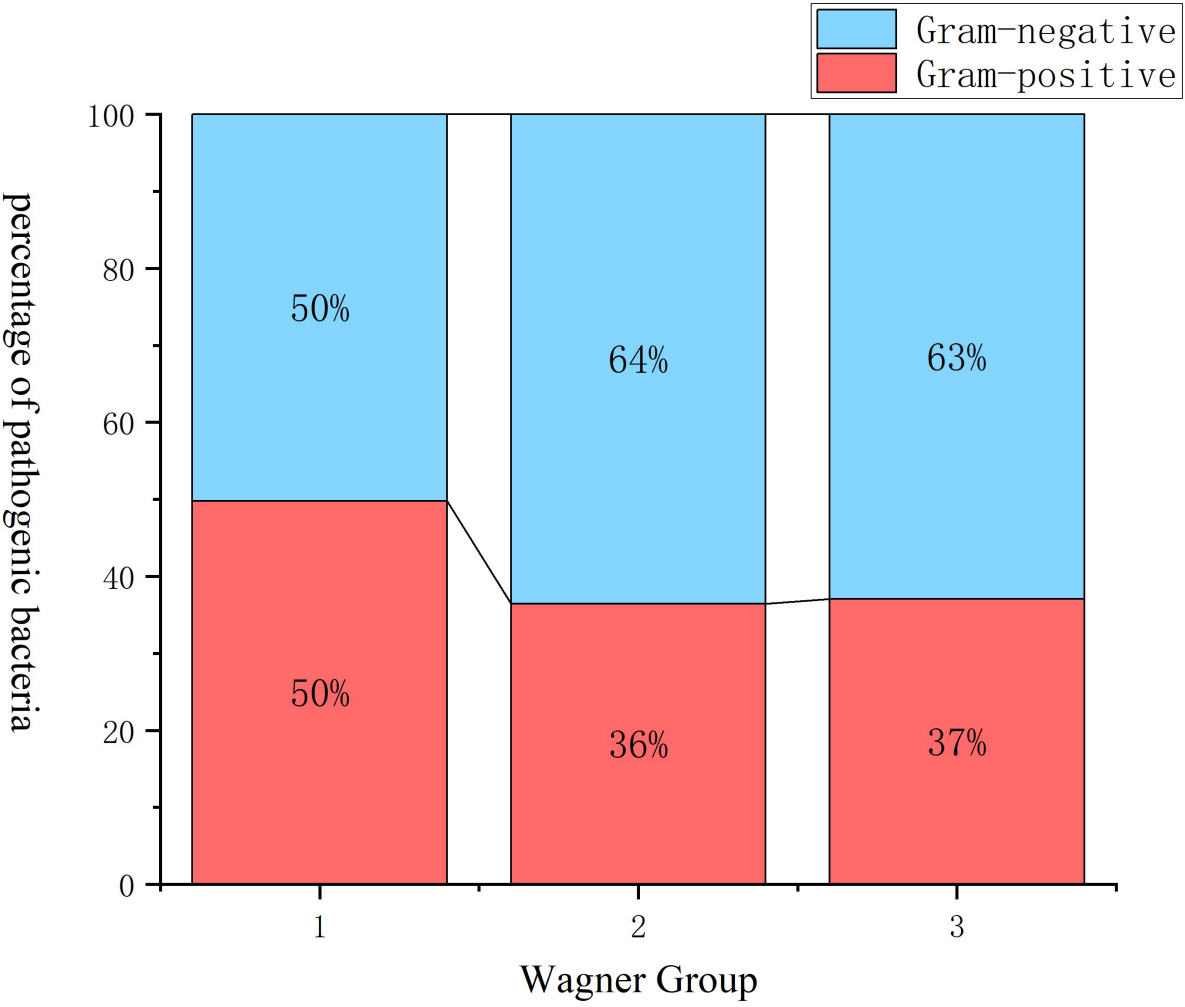
Gram-stain profiles of bacterial pathogens in DFI patients stratified by Wagner grades (excluding fungi). With the increase of Wagner grades, the proportion of Gram-negative bacteria was significantly higher than that of Gram-positive bacteria (p=0.026). Wagner group 1: Wagner grades 1-2, Mild-Moderate; Wagner group 2: Wagner grade 3, Severe; Wagner group 3: Wagner grades 4-5, Critical; DFI, diabetic foot infection.

After excluding fungal infections, statistical analysis was conducted on 434 pathogenic bacterial strains according to Wagner grades, and the chi-square test was refined (p=0.026). The results showed that with the increase in Wagner grade, the proportion of Gram-negative bacteria was significantly higher than that of Gram-positive bacteria (**Figure 5**).

### 3.5 Characteristics of the SII in patients across different UACR and Wagner grades

The data for the three UACR and Wagner groups did not follow a normal distribution (p < 0.001). Non-parametric tests revealed significant differences among the groups (p < 0.001). Further analysis with independent sample tests demonstrated that the SII in the UACR <30 mg/g group was significantly lower than the other two groups (p = 0.005 and p < 0.001), while no significant difference was observed between the 30 ≤ UACR <300 mg/g and UACR ≥300 mg/g groups. In contrast, the Wagner grade 3 group exhibited a significantly higher SII compared to the other two groups (p < 0.001 and p = 0.017). Additionally, non-parametric tests indicated that the SII was significantly higher in polymicrobial infections than in single bacterial infections (p = 0.003), but no significant differences were found in bacterial staining or drug resistance profiles.

## 4 Discussion

The present study, based on retrospective cohort data from a tertiary hospital in northern China between 2020 and 2023, focuses on the association between the UACR and both the pathogen spectrum and inflammatory markers in patients with DFI. It systematically reveals the interactions between DN and DFI at the levels of microbial distribution and inflammatory response, thereby providing evidence-based support for the stratified diagnosis and treatment of DFI.

In our study, the patient population was predominantly male (64.63%), which aligns with findings from previous studies (12). This demographic trend may be attributed to inadequate attention to foot care and gender-associated differences in lifestyle or occupational environments. The cohort primarily comprised middle-aged and elderly diabetic patients, who exhibited impaired ability to manage blood glucose, lipid levels, and blood pressure, resulting in a significantly elevated risk of diabetic foot complications and related comorbidities (13). These findings underscore the necessity for intensified follow-up protocols and targeted patient education to implement timely preventive measures, thereby reducing the incidence and progression of diabetic foot complications and associated conditions in this vulnerable population.

The diversity of bacterial infections poses a major challenge in the treatment of diabetic foot complications. Diabetes mellitus compromises the immune system by impairing the phagocytic capacity of mononuclear macrophages (14) and reducing the recruitment efficiency of granulocytes (15). Without timely administration of appropriate antibiotics and debridement of infected tissues, localized infections may worsen, potentially progressing to systemic complications such as septic shock, multi-organ failure, and death. Antimicrobial susceptibility testing of bacteria is an important basis for clinical antibiotic selection, yet the testing process is usually time-consuming (taking 2 to 3 days), resulting in delayed reporting of results. Owing to the inability to obtain timely results and the limited accuracy of the test, it is difficult to provide prompt and effective guidance for the initial empirical selection of antibiotics for DFI patients with urgent clinical treatment needs. Prior to the availability of culture results, antibiotic selection mainly relies on clinicians’ experience and the severity of infection (16).

Stratified differences in pathogen distribution are one of the core findings of the present study. Among the 325 patients with confirmed bacterial infections, a total of 447 pathogenic bacterial strains were isolated. The majority of infections were attributed to Staphylococcus aureus (21.80%). Although S. aureus monoinfections accounted for a high proportion in the microalbuminuria (30 ≤ UACR <300 mg/g) and macroalbuminuria (UACR ≥300 mg/g) groups, the overall predominant pathogens were Gram-negative bacteria, including Proteus mirabilis and Escherichia coli. Previous studies have indicated that Gram-negative bacteria have now replaced Gram-positive bacteria as the predominant pathogenic bacteria not only in northern China, but also in southern and central China (17). A similar shift in the pathogenic spectrum of diabetic foot infections—with Gram-negative bacteria emerging as the primary pathogens—has also been observed in some developing countries (18, 19). In contrast, Gram-positive bacteria (primarily S. aureus) dominated the normoalbuminuria (UACR <30 mg/g) group.

Among the 447 strains, the multidrug resistance rate(MDR) (16%) was notably lower than the 57% reported nationally in China (20).This may be because patients in this study are older, pay less attention to foot infections, and use fewer antibiotics, so pathogens are more sensitive to antibiotics after admission. The primary resistant strain identified was methicillin-resistant Staphylococcus aureus(MRSA). The observed methicillin resistance rate is hypothesized to correlate with widespread inappropriate antimicrobial use (e.g., self-medication and over-the-counter practices) and high population density, which facilitates rapid dissemination of multidrug-resistant organisms (21).In this study, MRSA accounted for 23% of all detected Staphylococcus aureus isolates. The disease duration of patients infected with this resistant strain was close to the cohort average (12.44 ± 2.09 months). Most of these patients presented with Wagner grade ≥3 and UACR >30 mg/g, and over half had a DFI disease course of less than 1 month. The high pathogenicity and virulence of MRSA may contribute to the rapid onset and progression of DFI. The genetic adaptability of MRSA and the emergence of successful epidemic clones continue to pose a major threat to global health (22). Consequently, prevention and treatment of S. aureus infections must not be overlooked. Given that the skin is the most common colonization site for S. aureus, prompt intervention is critical once DFI occurs; otherwise, delayed management may significantly worsen clinical outcomes. Recent studies have reported a progressive increase in infections caused by extended-spectrum β-lactamase (ESBL)-producing bacteria (23). Among the detected Gram-negative pathogens, Escherichia coli exhibited the highest proportion of ESBL-producing strains (43%), necessitating careful selection of appropriate antibiotics in clinical practice. Additionally, the rate of polymicrobial infections (32%) was lower than previously reported (54%) (24). Notably, the microalbuminuria group (30 ≤ UACR <300 mg/g) demonstrated a higher polymicrobial infection rate compared to the other two groups. Most studies indicate that DFI frequently present as polymicrobial infections (18, 24), the therapeutic challenges they pose warrant continued attention. Empirical antimicrobial therapy, particularly when comprehensive pathogen identification is unavailable (25), should integrate UACR levels and clinical manifestations to optimize treatment strategies. For antibiotic selection, when the urinary albumin-to-creatinine ratio (UACR) is < 30 mg/g, infections are predominantly caused by Gram-positive bacteria, with Staphylococcus aureus being the most prevalent pathogen. Empirical antibiotic therapy should prioritize agents active against Gram-positive bacteria, such as semi-synthetic penicillinase-resistant penicillins and first-generation cephalosporins. For patients with a β-lactam allergy, clindamycin or fluoroquinolones may be an alternative. In the 30 ≤ UACR < 300 mg/g group, the rate of polymicrobial infection is the highest and Gram-negative bacteria are the dominant pathogens; thus, combination anti-infective regimens should be emphasized. Options include β-lactam/β-lactamase inhibitors and second/third-generation cephalosporins. For patients who have recently received antibiotics with poor therapeutic efficacy, the regimen may be escalated to potent β-lactamase inhibitors (e.g., piperacillin-tazobactam) or carbapenems, with close attention paid to ESBL-producing strains. When UACR ≥ 300 mg/g, Gram-negative bacteria remain the predominant pathogens and levels of inflammatory markers (e.g., ESR, CRP) are the highest. Antibiotic selection should focus on potent coverage of Gram-negative bacteria, with careful dosage adjustment to avoid nephrotoxicity. Additionally, vancomycin or linezolid should be considered for patients with a history of MRSA exposure.

The Wagner ulcer scale is a widely used scale for assessing wound severity, particularly in patients with DFI.In the present study, the correlation analysis of Wagner classification with UACR further reveals the interaction between the progression of DN and DFI.A significant association was observed between Wagner grades and bacterial distribution (p=0.04). At Wagner grade 1, the infection rates of Gram-positive and Gram-negative bacteria were comparable. However, as the Wagner grade increased, the proportion of Gram-negative bacteria became significantly higher than that of Gram-positive bacteria, and the disease duration of diabetic foot (DF) lesions progressively lengthened. Regarding the relationship between Wagner grades and UACR, the number of Wagner grade 1 patients decreased with rising UACR levels, whereas the number of Wagner grade 3 patients increased with elevated UACR. Combined with our previous analysis of the distribution of UACR results, deterioration of renal function and DFI may interact and exacerbate each other through multiple mechanisms: On the one hand, the accumulation of advanced glycation end products (AGEs) in a hyperglycemic environment activates the AGEs-receptor for advanced glycation end products (RAGE) axis, inducing the production of reactive oxygen species (ROS) and a vicious cycle of oxidative stress and inflammation (26). This cycle not only aggravates glomerular injury and promotes the progression of proteinuria, but also exacerbates foot neuropathy and vascular sclerosis, reduces skin resistance to infection (27), and ultimately forms a vicious cycle of “DN progression-DFI exacerbation-increased inflammation-DN deterioration”. On the other hand, the present study found that ALB levels were negatively correlated with UACR levels. Urinary protein loss caused by DN is a major factor leading to decreased serum albumin levels; meanwhile, wound exudation in DFI also results in albumin loss, and frequent debridement further exacerbates albumin consumption. Given that albumin is a key substance for maintaining immune cell function and wound repair, hypoalbuminemia impairs the phagocytic capacity of neutrophils and reduces fibroblast proliferation, thereby delaying wound healing. This often traps patients in a vicious cycle of “hypoalbuminemia - recurrent infection - refractory wound healing” (28).

Analysis of inflammatory markers provides a new perspective for the evaluation of DFI complicated by DN. Traditional inflammatory markers are positively correlated with UACR, and ESR has superior diagnostic value to the other markers in the evaluation of DFI complicated by DN. This is consistent with the finding reported by Guo et al. that there is a significant association between ESR and DN in patients with type 2 diabetes mellitus (T2DM) (29).ESR is a direct manifestation of the “bidirectional exacerbation” of the inflammatory response between DN and DFI. Additionally, ESR has greater diagnostic value for osteomyelitis (30), especially in patients with Wagner Grade 4–5. Moreover, compared with PCT and CRP, ESR can simultaneously reflect both infectious inflammation and non-infectious inflammation. SII is a novel composite inflammatory biomarker. The SII is calculated using the formula: SII=(Platelet count×Neutrophil count)/Lymphocyte count. Numerous studies have established associations between SII and diabetes-related complications, including Diabetic macular oedema (DME) (31),DN (32) and diabetes depression (33). Compared to traditional inflammatory markers, SII integrates three immune cell types, providing a comprehensive reflection of systemic inflammation and demonstrating superior prognostic value (34).However, current research lacks analyses on the relationship between the SII and DFI or DN. Our study revealed that the SII in the normoalbuminuria group (UACR <30 mg/g) was significantly lower than in the other two groups, while the SII in Wagner group 3 patients was markedly elevated compared to the other two groups. A higher SII indicates a poorer prognosis for DN (28).Compared with other inflammatory markers, the SII showed no significant difference in performance. Although previous studies have confirmed that SII can serve as a diagnostic criterion for DN, our study found that SII cannot accurately predict disease severity. Nevertheless, its advantages—including low cost, ease of detection, and more comprehensive integration of foot inflammation-related information—remain indispensable in the process of rapid disease diagnosis (35).

Based on the aforementioned study results, the present study has clear guiding significance for the clinical management of DFI complicated by DN. It is recommended that during clinical treatment, the UACR and Wagner classification be routinely detected in DFI patients at admission. The pathogen type can be preliminarily determined through the combined assessment of these two indicators: for instance, if UACR < 30 mg/g and Wagner classification is Grade 1–2, priority should be given to gram-positive bacterial infection; if UACR ≥ 30 mg/g and Wagner classification is Grade ≥ 3, vigilance should be paid to gram-negative bacterial infection and polymicrobial infection. In terms of inflammatory monitoring, regular detection of the ESR should be conducted, and dynamic assessment of the effectiveness of inflammation control should be performed. Meanwhile, individualized nutritional support regimens should be developed for patients with high UACR levels, with supplementation of high-quality protein (such as albumin, lean meat, and fish) to improve hypoalbuminemia and break the vicious cycle of “inflammation – hypoalbuminemia – infection”. In terms of the prevention and control of drug-resistant bacteria, screening of patients infected with MRSA and ESBL-producing bacteria should be enhanced to avoid cross-infection. Meanwhile, antibiotic regimens should be adjusted strictly in accordance with the results of drug sensitivity tests to prevent the spread of drug resistance caused by irrational drug use.

Our analysis of bacterial infections, related inflammatory biomarkers, and the SII revealed that elevated inflammatory biomarkers correlate with greater urinary protein loss, indirectly indicating poorer renal prognosis. In the context of DFI, higher inflammatory biomarker levels reflect more severe foot pathologies and an increased likelihood of polymicrobial infections, suggesting that patients with advanced Wagner-grade DFI experience intensified local and/or systemic inflammatory responses. Therefore, for patients with concurrent DN and DFI, clinical management should prioritize timely adjustment of antibiotic regimens and local debridement, alongside a tailored dietary plan rich in high-quality protein and low in refined carbohydrates. This dual approach ensures adequate nutritional support while maintaining optimal glycemic control, thereby preserving renal function and accelerating foot wound healing.

However, the current study has several limitations. This study was designed as a single-center, retrospective cross-sectional observational study, which has potential selection and information biases and is unable to reveal the causal relationship and dynamic changes between UACR and DFI. Microbial detection was performed via cotton swab culture of ulcer secretions, which cannot fully reflect the pathogen spectrum of deep infections and is subject to bias from superficial flora interference. Although a variety of confounding variables were adjusted for in the regression analysis, unrecognized confounding variables may still exist (e.g., hospitalization status, ulcer nursing care, etc.). In addition, the enrolled cases of DFI in this study were predominantly mild, which limits the generalizability of the research findings. As a preliminary exploratory study, this work only provides a reference for the potential trends of different microbial infections in clinical practice. Meanwhile, the inflammatory marker data were all from single-time-point measurements and cannot reflect their dynamic changes over time. Future studies should refine animal models and adopt advanced pathogen identification methods such as metagenomic sequencing to elucidate the interactions between the UACR, inflammatory markers and pathogens in wound infections. Furthermore, longitudinal studies need to be conducted and data collection protocols optimized to dynamically monitor the associations between inflammatory markers and the progression of DN and DFI.

## 5 Conclusion

This retrospective study analyzed the characteristics of pathogenic bacteria and UACR in patients with DFI at a tertiary hospital in northern China. By stratifying UACR levels, we aimed to delineate the local distribution of DFI pathogens, providing clinicians with evidence to guide empirical antimicrobial therapy. UACR >30 mg/g is associated with an increased risk of Gram-negative/polymicrobial DFI, thus prompting empirical coverage for these pathogens in patients with Wagner grade 3–5 ulcers. In addition to aggressive infection management, DFI treatment should emphasize monitoring UACR and assessing systemic inflammatory status. Therapeutic strategies must prioritize agents that concurrently address both conditions to prevent their mutual exacerbation and break the vicious cycle. Furthermore, this study contributes novel epidemiological data for regional and national healthcare planning and offers fresh insights into the interplay between DFI and DN for the global medical community. Future studies can expand the research population and improve long-term follow-up to dynamically monitor the temporal relationships between changes in UACR, inflammatory markers (such as SII and ESR) and pathogens. Meanwhile, basic experiments can be improved to evaluate the synergistic effects of drugs in improving UACR, reducing inflammation and promoting wound healing.

## Data Availability

The raw data supporting the conclusions of this article will be made available by the authors, without undue reservation.
